# Correlation between pain and depressive symptoms in patients with confirmed endometriosis during COVID-19 pandemic

**DOI:** 10.1007/s00404-023-07295-z

**Published:** 2023-12-16

**Authors:** Martina Helbig, Nora K. Schaal, Johannes Drumm, Flurina Fürst, Lisa Reinhart, Tanja Fehm, Ines Beyer

**Affiliations:** 1 Clinic for Gynecology and Obstetrics, University Hospital, Moorenstraße 5, 40225 Düsseldorf, Germany; 2 Department of Experimental Psychology, Heinrich-Heine-University, Düsseldorf, Germany; 3 Medical Faculty, Heinrich-Heine-University, Düsseldorf, Germany; 4 Academic Teaching Hospital Leverkusen, University of Cologne, Cologne, Germany

**Keywords:** Endometriosis, COVID 19, Pain, Depressive symptoms

## Abstract

**Background:**

Endometriosis is a chronic, estrogen-dependent, inflammatory condition which affects women of reproductive age physically and psychologically in their everyday life. The most common symptom is chronic lower abdominal pain. Apart from physical pain, endometriosis often also leads to an unfulfilled desire to give birth. In general, these two main aspects alone lead to emotional stress for patients and often initiate depressive symptoms. To what extent endometriosis patients are additionally affected by the COVID pandemic and its effects is to be determined in this study.

**Methods:**

Patients who presented at our endometriosis center and met the study criteria were offered participation in the study. A link to an online questionnaire (SoSci-Survey) was sent by email. The online questionnaire evaluated depressive symptoms before and during the pandemic as well as the pain perception and perceived support during the pandemic. The data of 167 fully completed questionnaires were evaluated and analyzed using SPSS.

**Results:**

The analysis of the questionnaires revealed a significant association between pain levels and depressive symptomatology in endometriosis patients during the pandemic. Patients with more severe pain showed significantly higher depressive symptoms than patients with little or no pain. During the pandemic, patients showed higher depressive symptoms than before. In addition, it was found that those endometriosis patients who felt left alone with their pain due to the consequences of the COVID pandemic, or who felt they had to endure the pain alone, also had higher depressive symptoms.

**Conclusion:**

In summary, it can be observed that endometriosis patients with a high pain burden had significantly higher depressive symptoms during the COVID pandemic. The consequences of the pandemic often led to the feeling of having to cope with the symptoms alone or having to endure pain alone, which in turn increased the depressive symptoms. As treating physicians, we should be aware of these connections and try to counteract them with targeted offers and support.

**Supplementary Information:**

The online version contains supplementary material available at 10.1007/s00404-023-07295-z.

## What does this study add to the clinical work

**Table:** 

Our results show that there was a significant correlation between pain and depressive symptoms in endometriosis patients during the COVID-19 pandemic. As treating physicians, we should be aware of these connections under special circumstances (e.g., the COVID pandemic) and try to counteract them with targeted offers and support.	

## Introduction

Endometriosis is a chronic, estrogen-dependent, inflammatory condition, which affects women. Endometrial-like mucosa spreads outside the uterus, predominantly in the pelvis. Etiology still remains unsolved, and a definitive diagnosis requires laparoscopy and histological proof. Severity of endometriosis is classified into stages, but although widely used, this system correlates poorly with symptom severity or reproductive prognosis [1, 2]. Even significant endometriosis lesions may be asymptomatic. Therefore, prevalence estimates are imprecise but, in high-income countries, range from 1 to 2% in populations of women of reproductive age [3]. Treatment options are limited to analgesics and hormonal suppression of ovarian function, which may be associated with side effects. Lesions removed surgically commonly recur [4], although ongoing medical suppression can prevent or reduce recurrence.

Endometriosis and its symptoms might affect psychological and social functioning of patients [5–7]. Therefore, endometriosis is considered a disabling condition that may significantly compromise affected women’s relationships, sexuality, and mental health [6, 8, 9]. Endometriosis is a complex disease, where psychological factors have an important role in determining the severity of symptoms and the effectiveness of the treatments. According to recent data, women with endometriosis are at risk for anxiety, depressive symptoms, and other psychiatric disorders [10].

The quality of life of women suffering from endometriosis is compromised in a variety of aspects [3, 11]. Besides physical disadvantages and challenges, the patients must face possible psychological burdens which might affect their social interactions and relationships. As a result, women with the disease often face different types of stress, including thoughts of surgery, possible complications (e.g., unwanted childlessness), and long-term effects on their health/performance.

In March 2020, the WHO declared the increasing appearance of COVID-19 to be a global pandemic, which drastically transformed our daily lives. Self-isolation practices—enforced to flatten the curve of COVID-19—complicated the availability and accessibility of medical care worldwide. Various restrictions and regulations increased stress and anxiety and as a result, many counseling appointments and treatments were postponed for a prolonged period of time or even canceled altogether. All the while the burden of endometriosis remained.

Women with endometriosis generally face chronic pain, which was aggravated during the pandemic by higher levels of anxiety, social restrictions, and fears about COVID-19 itself. Women with the desire to have children were additionally worried about delays in receiving services due to the cancelation of elective surgery and the reduced availability of consultation services [3, 12].

This study focusses on the question of how the COVID-19 pandemic and its consequences may have affected patients’ physical and psychological well-being in addition to (pre-existing) endometriosis-related symptoms and burdens. In particular, we want to investigate whether our patients showed varying perception of pain and depressive symptoms before and during the COVID-19 period.

## Methods

This study is a monocentric prospective study. A total of 167 patients, aged 18 years or older, were included by having a complete data set. A list of possible participants was created by going through the list of patients who presented to the Clinic of Gynecology and Obstetrics of the University Hospital of Düsseldorf with already confirmed endometriosis or whose endometriosis was confirmed in the course of time. Patients were contacted by email or telephone and informed about this study. Recruiting took place from August 2021 until June 2022. After consent was given, an email containing a link to the survey was provided. Subsequently, participants gave informed written consent at the beginning of the online survey. Time for completion was approximately 20 min.

A questionnaire was developed after literature review (supplemental material). It was partially based on the validated quality of life and depression questionnaires, adjusted to the study setting. Its purpose was to evaluate physical and psychological parameters in patients suffering from endometriosis during the COVID-19 pandemic. First, demographics and basics regarding the patient’s endometriosis (date of first symptoms, date of diagnosis, current or backdated treatment) were evaluated. Academic degree was evaluated asking whether the patients have a university degree. Then we asked the women about their quality of life in general and their strategies to improve it, both before and during the COVID-19 pandemic (suggestions for selection). Subsequently, we determined how participants experienced their pain load before and during the COVID-19 pandemic and whether they had the feeling of being alone with their pain (visual analog scale). Depressive symptoms and depression levels before and during the pandemic were evaluated using a modified validated questionnaire.

### Participants

**Fig. 1:**
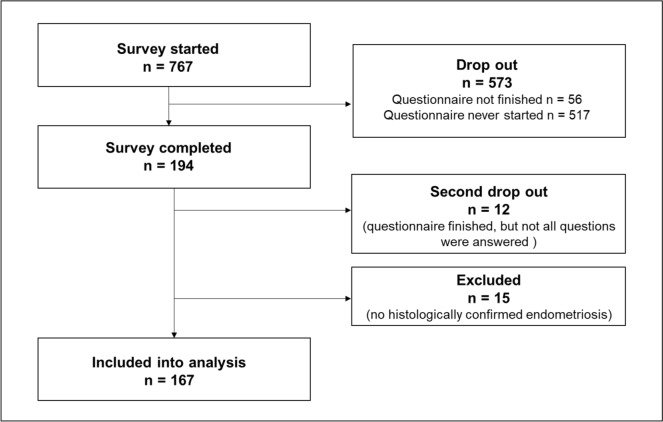
Flowchart of participants

Overall, data from 167 women who had a complete data set were included in this study (Fig. 1). The average age of participants was 31 $\pm$ 7 years. During the study period, a total of 767 women were asked via email to participate in the study. Five hundred seventeen women never started the questionnaire. Fifty-six patients started but did not finish the questionnaire. One hundred ninety-four women in total finished the questionnaire, twelve of whom did not complete all the sections. Finally, 15 patients were ruled out based on other exclusion criteria.

### Statistical analysis

The analysis was performed using IBM SPSS ® Version 27. Two timepoints were classified: before and during the COVID-19 pandemic. Participants were categorized into two groups, those without pain and those with pain. Women who indicated similar, less, or no pain were categorized as the ‘no pain perception group’ and women indicating pain or even more pain were classified as the ‘pain perception group’ (pain yes/no).

First of all, the two groups (pain yes/no) were compared on demographic valuables using independent sample *t* tests. In addition, we performed Chi-square test to check whether the groups differ regarding academic status.

Then a 2 × 2 mixed factorial ANOVA was performed with a within-subject factor *time* (before vs. during COVID-19 pandemic) and the between-subject factor *group* (patients with or without pain perception). To further disentangle the significant interaction, independent sample *t* tests for each timepoint were applied. Furthermore, we repeated the 2 × 2 mixed factorial ANOVA with age as a co-variate.

In addition, correlation analyses were performed between the variables that endometriosis patients “felt left alone with their pain due to the consequences of the COVID-19 pandemic” or “felt they had to bear the pain solely” and depressive symptoms.

## Results

The data of 167 women were complete. Fifty-four were in the group with pain perception and one hundred thirteen were in the group without pain perception.

First of all, the two groups (pain yes vs. no) were compared on demographic valuables to see whether the groups differ. The results show that there is a significant difference in age (*p* = 0.009). The non-pain group is older (32.8 ± 7.1 years) than the pain group (29.7 ± 6.3 years); however, on academic status, no difference was shown (*p* = 0.123).

**Fig. 2:**
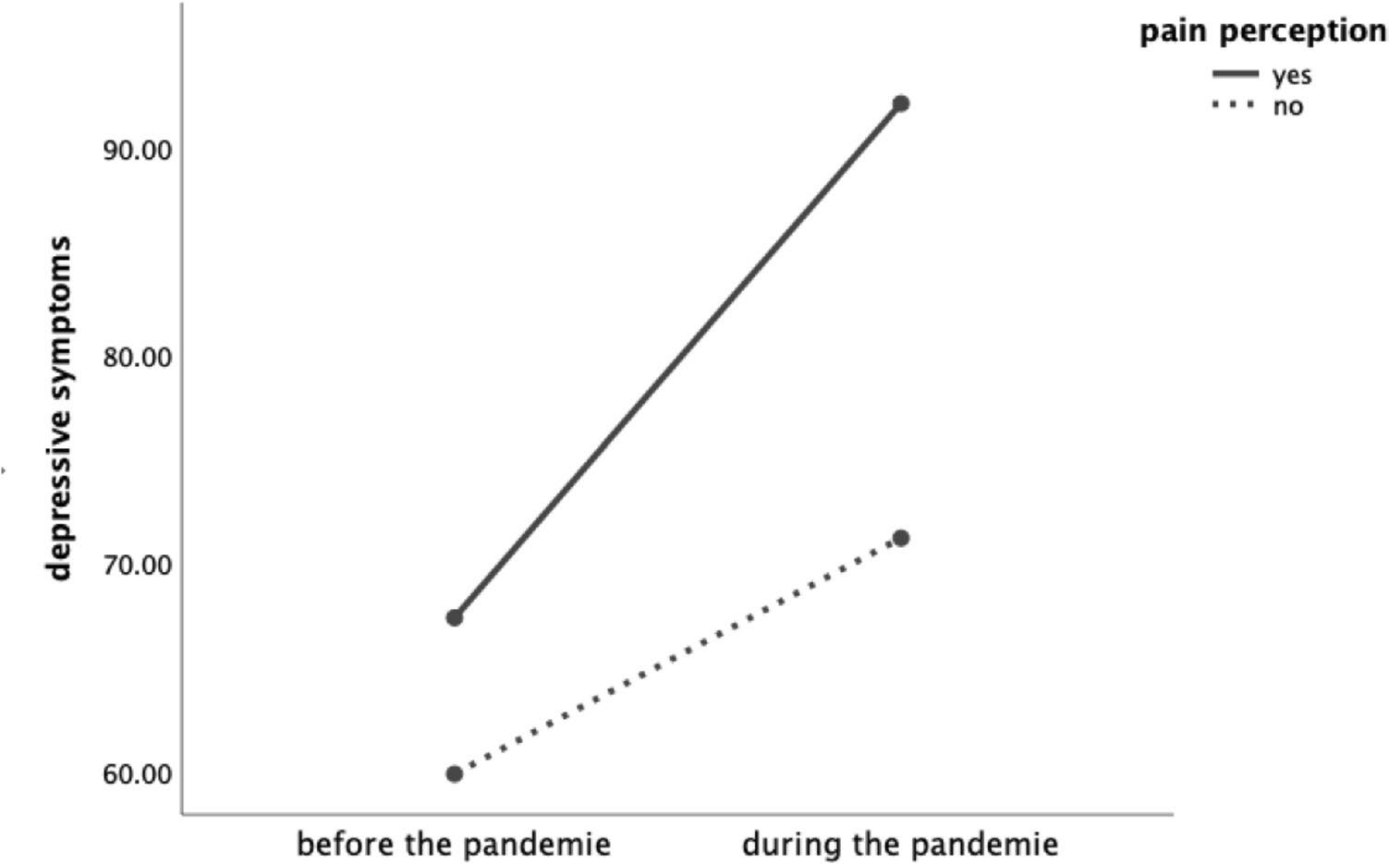
The two groups (pain perception yes vs. no) did not differ regarding their depressive symptoms before the pandemic. However, during the COVID-19 pandemic, the women with acute pain showed significant higher depressive symptoms than the group of women with no pain

The 2 × 2 mixed factorial ANOVA revealed a significant main effect of *time*, *F* (1,165) = 115.70, *p* < 0.001, a significant effect of group, *F* (1, 165) = 9.16, *p* = 0.003, and a significant time*group interaction, *F* (1,165) = 15.98, *p* < 0.001. The main effect of *time* indicates that women reported more depressive symptoms during the pandemic (*M* = 77.71 ± 31.70) than before the pandemic (*M* = 62.19 ± 29.51). The main effect of *group* shows that women with pain perception overall report higher depressive symptoms (*M* = 79.74 ± 31.46) than the group of women without pain perception (65.52 ± 29.04). Furthermore, the post hoc independent-samples *t* tests revealed that the two groups (pain yes vs. no) did not differ regarding their depressive symptoms before the pandemic, t (165) = 1.53, *p* = 0.128. However, during the COVID-19 pandemic, the women with acute pain showed significant higher depressive symptoms (*M* = 92.10 ± 30.98) than the group of women with no pain (*M* = 71.20 ± 29.95), *t* (165) = 4.13, *p* < 0.001 (Fig. 2).

The 2 × 2 mixed factorial ANOVA was repeated with age as a co-variate to check the influence. However, the result pattern was the same showing that age did not influence effect of pain on depressive symptoms.

**Fig. 3:**
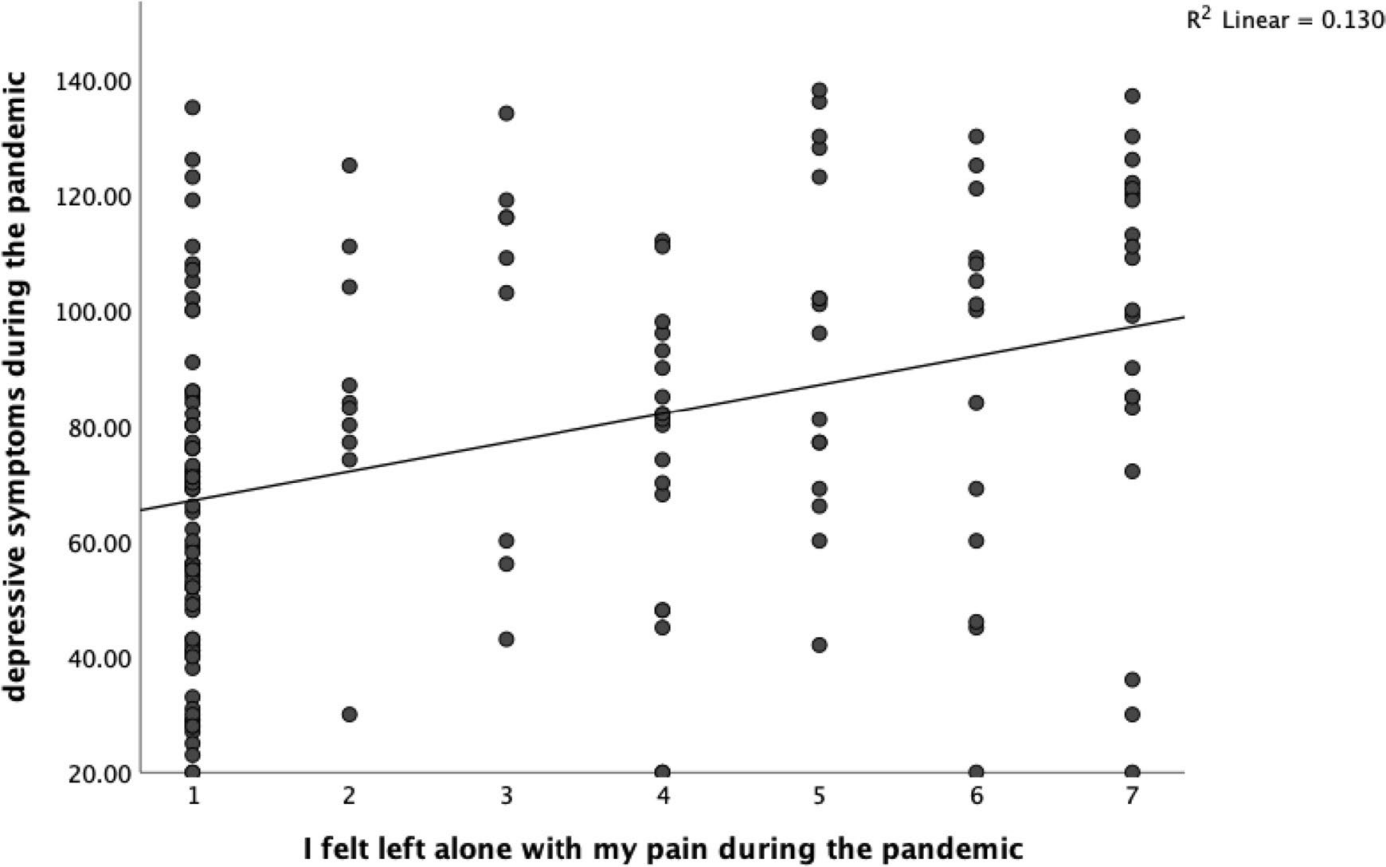
Linear correlation of depressive symptoms during the COVID-19 pandemic and the feeling of being left alone with the pain

**Fig. 4:**
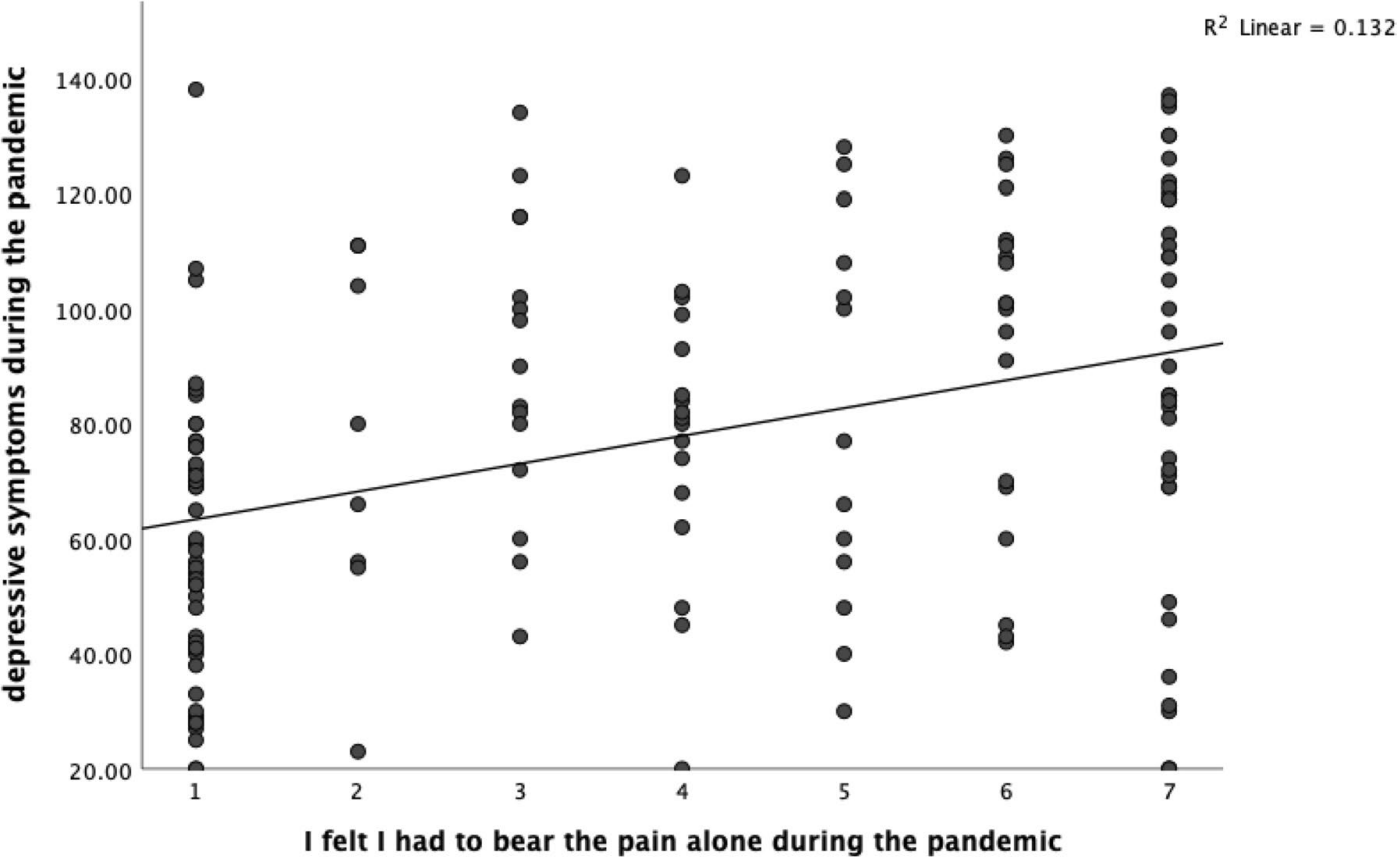
Linear correlation of depressive symptoms during the COVID-19 pandemic and the feeling of having to bear the pain alone

In addition, the correlation analysis revealed significant positive associations between the variables that endometriosis patients felt left alone with their pain due to the consequences of the COVID-19 pandemic, or felt they had to bear the pain alone, and depression symptoms (both *p* values < 0.001). The linear correlations are shown in Figs. 3 and 4.

## Discussion and conclusion

Aim of this study was to investigate whether the additional factor of the COVID-19 pandemic and its consequences might have affected patients’ physical and psychological well-being in addition to (pre-existing) endometriosis-related symptoms and burdens. We were especially curious on how the COVID-19 pandemic influenced pain perception and depressive symptoms, as well as the correlation of both, in patients with endometriosis.

Our results show that there is a significant correlation between pain and depressive symptoms in endometriosis patients during the COVID-19 pandemic. All our participants showed an increased level of depressive symptoms during the COVID-19 pandemic. In addition, patients with more severe pain indicated significantly higher depressive symptoms than those with little or no pain during the COVID-19 pandemic. As the two groups differed regarding age, this is a factor which should be considered in the future. However, our results show that this does not influence the effect of pain status on depressive symptoms. An assumption on why the ‘non-pain group’ may be older than the ‘pain group’ could be that patients adapt to their pain levels with age, and therefore older patients less often report pain regarding endometriosis than younger patients. Furthermore, our study shows that endometriosis patients who felt left alone with their pain due to the consequences of the COVID-19 pandemic, or who felt they had to bear the pain alone, also scored higher on the depression scale.

It is well known that endometriosis is a very complex disease and psychological factors have an important role in determining the severity of symptoms and the effectiveness of the treatments [10]. According to the literature, women with endometriosis are at risk for anxiety, depressive symptoms, and other psychiatric disorders [10, 13–15]. A meta-analysis of 24 studies by Gambadauro et al. [16] has shown higher levels of depression among women with endometriosis compared to controls. In addition, the review points out that endometriosis patients reporting pelvic pain had significantly higher levels of depression compared to those without pain. Despite the fact that to date it still remains unclear if psychological diseases are a result of endometriosis itself or other factors such as chronic pelvic pain, the connection is well known and documented in literature [5, 10, 13–16].

Regardless of whether exacerbated by endometriosis or not, the COVID-19 pandemic and its restrictions may trigger negative psychological effects [12, 17] in patients with endometriosis, which require diverse and multimodal strategies [18]. Furthermore, it has been reported that patients with endometriosis experience high levels of stress due to the negative impact of the endometriosis‐related symptoms on all aspects of life, including work, relationships, and fertility [19]. According to Graham et al. [20], patients with endometriosis reported higher levels of stress and higher depression scores, especially those with severe endometriosis. In addition, a Turkish survey has indicated that patients with endometriosis were afraid of experiencing endometriosis‐related problems during the pandemic period [21]. Arena et al. [12] have stated that COVID-19 pandemic significantly impacted the lives of women with endometriosis, who appeared to have a considerable risk of PTSD. Older age, higher anxiety levels, and unemployment were independently associated with the risk of developing PTSD.

Regarding the results of our study, it remains to be discussed whether severe pain due to endometriosis leads to higher depression scores or whether (pre-existing) depressive symptoms lead to higher pain perception, or both is the case, complicating each other.

Our study, although with a little sample size, supports previous findings regarding the correlation of pain level and depressive symptoms in the circumstances of the COVID-19 pandemic [20]. Due to our sample size, we have to keep in mind that our data might not be representative for the overall German population. Another limitation, which should be mentioned here, is that the applied questionnaire used self-created items to evaluate pain perception and quality of life of endometriosis patients instead of using validated questionnaires. However, as we were interested in the interactions of endometriosis-related symptoms and COVID-19 restrictions, no validated questionnaires existed at the time of our survey.

In addition, we would like to mention that another interesting fact for future studies would be to evaluate the relationship status and partnership of our patients to find out how that might have an impact as well [22]. Along with Schick et al. [22], the impact of the partner should be taken into account when counseling or treating women with endometriosis. Their study has shown a high interdependence and reciprocal influence from both partners—positively and negatively—concerning psychological distress and sexual satisfaction. The authors state that the impact of endometriosis on the partner has probably been underestimated [22]. It is conceivable that the impact of a partnership can interfere—positively and negatively—with the well-being and mental situation of endometriosis patients, especially under the circumstances of the COVID-19 pandemic.

As treating physicians, we must be aware of and try to better understand the relationship between endometriosis and psychological diseases. During the COVID-19 pandemic, the quality of life of endometriosis patients seems to have decreased due to multiple factors. We want to emphasize the importance of a multidisciplinary approach in the management of women with endometriosis. Furthermore, psychological assessment should be provided to identify women at risk of developing symptoms of anxiety and depression and provide them an adequate psychological support [10]. Furthermore, there ought to be more awareness for the psychosocial impact of endometriosis, especially in regard to social support and understanding [22].

High-quality telehealth might be a possibility for shorter and less complex appointments and may continue to be used alongside face-to-face appointments according to patient preference [23].

Leonardi et al. have summarized various problem-focused and emotion-focused strategies that are non-medicational and non-surgical [18]. Patients should learn about the importance of self-care (e.g., physical exercise, stress reduction, alternative medical treatments and/or nutrition) and how this could help to promote patient self-efficacy. This in turn can help patients to feel less helpless and reliant on healthcare providers for relief [23]. These techniques are certainly also useful for patients now that the world is “back to normal”.

Our common goal is and remains to be the reduction of the impact of endometriosis and its multiple consequences on the quality of life and psychological well-being of patients.

### Supplementary Information

Below is the link to the electronic supplementary material.

Supplementary file1 (DOCX 214 KB)

## Data Availability

The dataset used for the current study is available from the corresponding author on reasonable request.

## References

[1] Vercellini P, Viganò P, Somigliana E, Fedele L (2014). Endometriosis: pathogenesis and treatment. Nat Rev Endocrinol.

[2] Ulrich U, Buchweitz O, Greb R, Keckstein J, von Leffern I, Oppelt P (2014). National German Guideline (S2k): guideline for the diagnosis and treatment of endometriosis: long version-AWMF Registry No. 015-045. Geburtshilfe Frauenheilkd.

[3] Rowe H, Quinlivan J (2020). Let’s not forget endometriosis and infertility amid the covid-19 crisis. J Psychosom Obstet Gynecol.

[4] Hickey M, Ballard K, Farquhar C (2014). Endometriosis. BMJ.

[5] Friedl F, Riedl D, Fessler S, Wildt L, Walter M, Richter R (2015). Impact of endometriosis on quality of life, anxiety, and depression: an Austrian perspective. Arch Gynecol Obstet.

[6] Zheng JS, Hua LJ, Hua SJ, Ran SP, He LJ (2012). Health-related quality of life in women with endometriosis: a systematic review. J Ovarian Res.

[7] Gruber TM, Mechsner S (2021). Pathogenesis of endometriosis: the origin of pain and subfertility. Cells.

[8] Vitale SG, La Rosa VL, Rapisarda AMC, Laganà AS (2017). Impact of endometriosis on quality of life and psychological well-being. J Psychosom Obstet Gynaecol.

[9] Siedentopf F, Tariverdian N, Rücke M, Kentenich H (2008). Arck PC 2008 Immune status, psychosocial distress and reduced quality of life in infertile patients with endometriosis. Am J Reprod Immunol N Y N 1989.

[10] Laganà AS, La Rosa VL, Rapisarda AMC, Valenti G, Sapia F, Chiofalo B (2017). Anxiety and depression in patients with endometriosis: impact and management challenges. Int J Womens Health.

[11] Bulletti C, Coccia ME, Battistoni S, Borini A (2010). Endometriosis and infertility. J Assist Reprod Genet.

[12] Arena A, Orsini B, DegliEsposti E, Raimondo D, Lenzi J, Verrelli L (2021). Effects of the SARS-CoV-2 pandemic on women affected by endometriosis: a large cross-sectional online survey. Ann Med.

[13] van Barneveld E, Manders J, van Osch FHM, van Poll M, Visser L, van Hanegem N (2022). Depression, anxiety, and correlating factors in endometriosis: a systematic review and meta-analysis. J Womens Health 2002.

[14] Wang Y, Li B, Zhou Y, Wang Y, Han X, Zhang S (2021). Does endometriosis disturb mental health and quality of life? A systematic review and meta-analysis. Gynecol Obstet Invest.

[15] Della Corte L, Di Filippo C, Gabrielli O, Reppuccia S, La Rosa VL, Ragusa R (2020). The burden of endometriosis on women’s lifespan: a narrative overview on quality of life and psychosocial wellbeing. Int J Environ Res Public Health.

[16] Gambadauro P, Carli V, Hadlaczky G (2019). Depressive symptoms among women with endometriosis: a systematic review and meta-analysis. Am J Obstet Gynecol.

[17] Brooks SK, Webster RK, Smith LE, Woodland L, Wessely S, Greenberg N, Rubin GJ (2020). The psychological impact of quarantine and how to reduce it: rapid review of the evidence. Lancet..

[18] Leonardi M, Horne AW, Vincent K, Sinclair J, Sherman KA, Ciccia D (2020). Self-management strategies to consider to combat endometriosis symptoms during the COVID-19 pandemic. Hum Reprod Open.

[19] Brasil DL, Montagna E, Trevisan CM, La Rosa VL, Laganà AS, Barbosa CP (2020). Psychological stress levels in women with endometriosis: systematic review and meta-analysis of observational studies. Minerva Med.

[20] Graham CJ, Brown SL, Vincent K, Horne AW (2020). International survey confirms that women with endometriosis-associated pain experience a high prevalence of pain imagery and coping imagery. Eur J Obstet Gynecol Reprod Biol.

[21] YalçınBahat P, Kaya C, Selçuki NFT, Polat İ, Usta T, Oral E (2020). The COVID-19 pandemic and patients with endometriosis: a survey-based study conducted in Turkey. Int J Gynecol Obstet.

[22] Schick M, Germeyer A, Böttcher B, Hecht S, Geiser M, Rösner S (2022). Partners matter: the psychosocial well-being of couples when dealing with endometriosis. Health Qual Life Outcomes.

[23] Evans S, Dowding C, Druitt M, Mikocka-Walus A (2021). I’m in iso all the time anyway: a mixed methods study on the impact of COVID-19 on women with endometriosis. J Psychosom Res Juli.

