# Supplementary material for: Correlation between pain and depressive symptoms in patients with confirmed endometriosis during COVID-19 pandemic
Source: Arch Gynecol Obstet. 2023 Dec 16;309(2):631–7. doi: 10.1007/s00404-023-07295-z (PMC10808472; doi:10.1007/s00404-023-07295-z)
Supplement: Supplementary file 1 — Supplementary file1 (DOCX 214 KB) [file 404_2023_7295_MOESM1_ESM.docx]

UKDendometrioseCOVID19 → base

**page 04**

# quality of life

**L117**

## please tick to what extent the statements apply to you

**L101**

0 = applies fully and completely

6 = does not apply

| 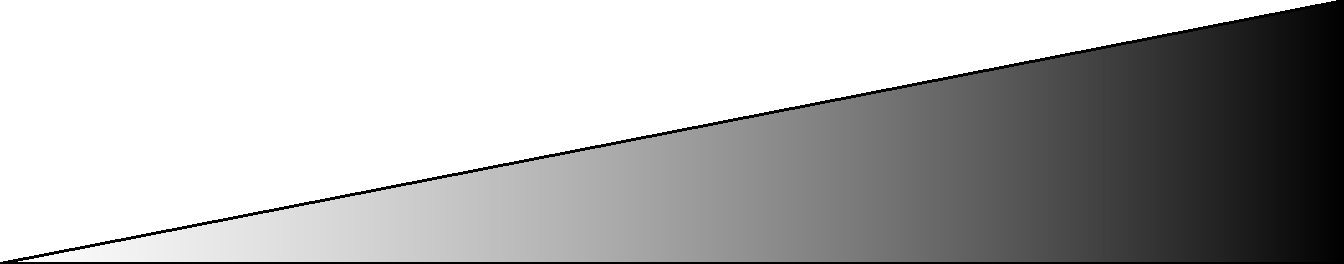 | | | | | | | |  |
| --- | --- | --- | --- | --- | --- | --- | --- | --- |
|  |  |  |  |  |  |  |  |  |
|  | 0 | 1 | 2 | 3 | 4 | 5 | 6 | n/a |
| I am concerned about, people I care about, might get sick with COVID 19 | 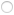 | 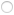 | 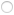 | 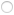 | 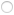 | 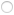 | 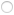 | 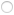 |
| I am concerned about I might get sick with COVID 19 | 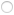 | 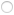 | 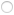 | 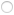 | 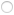 | 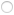 | 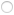 | 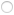 |
| Priorities of my life are different due to COVID 19 pandemic | 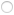 | 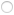 | 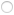 | 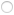 | 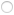 | 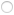 | 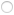 | 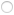 |
| Since COVID 19 pandemic I feel the pain more burdensome (if you had no pain, choose n/a) | 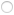 | 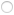 | 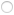 | 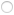 | 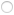 | 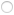 | 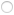 | 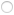 |
| COVID 19 pandemic made me realize that I am stronger than I thought I am | 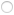 | 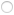 | 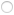 | 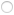 | 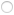 | 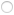 | 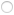 | 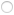 |
| Due to my endometriosis, I feel impaired compared to other (healthy) women | 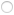 | 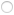 | 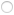 | 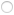 | 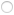 | 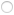 | 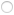 | 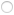 |
| During COVID 19 pandemic I feel even more impaired because of my endometriosis compared to other (healthy) women | 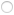 | 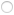 | 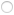 | 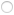 | 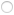 | 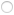 | 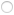 | 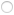 |
| Since COVID 19 pandemic I feel more stressed | 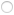 | 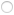 | 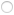 | 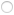 | 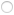 | 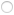 | 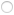 | 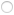 |
| I appreciate the support and sharing with my friends and family more now than I did before COVID 19 pandemic | 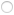 | 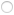 | 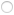 | 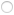 | 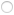 | 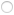 | 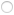 | 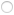 |
| Due to Corona, I feel impaired in my leisure time activities and thus also in my quality of life and perception of illness | 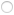 | 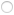 | 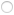 | 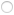 | 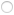 | 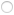 | 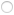 | 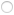 |
| I feel psychologically affected by Corona | 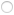 | 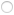 | 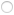 | 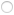 | 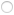 | 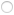 | 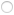 | 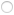 |
| I felt left alone with endometriosis due to COVID 19 pandemic | 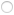 | 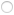 | 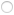 | 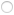 | 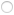 | 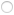 | 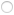 | 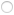 |
| I have the feeling that I receive less social support due to the Corona-related contact restrictions | 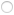 | 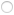 | 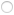 |  |  |  |  |  |
|  |  |  |  |  |  |  |  |  |

## before and during COVID 19

## please tick to what extent the following statements apply to you

0 = applies fully and completely

6 = does not apply

|  |  |  |  |  |  |  |  |
| --- | --- | --- | --- | --- | --- | --- | --- |
|  |  |  |  |  |  |  |  |
|  | 0 1 | 2 | 3 | 4 | 5 | 6 | n/a |
| Even before the pandemic, I often felt down and/or sad |  |  |  |  |  |  |  |
| During COVID 19 pandemic, I often felt down and/or sad |  |  |  |  |  |  |  |
| Even before the pandemic, I had less fun doing things that were actually fun, e.g. leisure activities |  |  |  |  |  |  |  |
| During the pandemic, I had less fun doing things that were actually fun, such as leisure activities |  |  |  |  |  |  |  |
| Even before the pandemic, I was less able to get excited about things that I normally find interesting. |  |  |  |  |  |  |  |
| During the pandemic, I was less able to get excited about things that I normally find interesting. |  |  |  |  |  |  |  |
| Even before COVID 19 pandemic, I felt low in motivation more often. |  |  |  |  |  |  |  |
| During COVID 19 pandemic, I felt low low in motivation more often. |  |  |  |  |  |  |  |
| Even before the pandemic, I was tired earlier and had trouble concentrating. |  |  |  |  |  |  |  |
| During the pandemic I was tired earlier and had difficulty concentrating |  |  |  |  |  |  |  |
| Even before the pandemic I thought worse of myself and felt less valuable |  |  |  |  |  |  |  |
| During the pandemic I thought worse of myself and felt less valuable |  |  |  |  |  |  |  |
| Even before the pandemic, I often had trouble getting up in the morning. |  |  |  |  |  |  |  |
| During the pandemic I had more difficulties getting up in the morning |  |  |  |  |  |  |  |
| Even before the pandemic, I felt bad and low in energy when I got up in the morning. |  |  |  |  |  |  |  |

During the pandemic, I felt bad and low in energy when I got up in the morning.

Even before the pandemic I was less able to work under pressure and perform well

During pandemic I was less able to work under pressure and perform well

Even before the pandemic, I often found it difficult to relax inwardly during periods of external rest.

During COVID 19 pandemic, I often found it difficult to relax inwardly during periods of external rest.

Even before the pandemic, I felt anxious and tense inside.

During the pandemic, I felt anxious and tense inside.

Even before the pandemic I was generally more emotionally irritable.

During the pandemic I was generally more emotionally irritable.

Even before the pandemic I felt anger and aggression more often.

During the pandemic, I felt anger and aggression more often.

Even before the pandemic, I often felt weak, helpless and overwhelmed.

During the pandemic, I often felt weak, helpless and overwhelmed.

Even before the pandemic I sometimes felt a numbness and inner emptiness.

During the pandemic I sometimes felt a numbness and inner emptiness.

Even before the pandemic, I was brooding a lot and my thoughts revolved around worries and predominantly negative topics.

During the pandemic I ruminated a lot and thoughts revolved around worries and predominantly negative issues.

Even before the pandemic, thoughts about my future made me pessimistic

During the pandemic, thoughts about my future made me pessimistic.

Even before the pandemic, increased dizziness with/without nausea and vomiting occurred.

During the pandemic, increased dizziness with/without nausea and vomiting occured.

Even before the pandemic, I was often plagued by fears.

During the pandemic, I was often plagued by fears.

Even before the pandemic I had massive anxiety/panic attacks.

During the pandemic I had massive anxiety/panic attacks.

**page 07**

# pain

**S009**

## use of painkillers before and during COVID 19 pandemic

1 = minimum of pain (e.g. mosquito bite)

10 = maximum of imaginable pain (e.g. worst teeth pain, giving birth)

0 1 2 3 4 5 6 7 8 9 10

n/a

Before the pandemic my average pain level was

During the pandemic my average pain level was

Before the pandemic, my strongest pain event was

During the pandemic, my strongest pain event was

## please choose to which extent the following statements apply

0 = applies fully and completely

6 = does not apply

It was harder for me to get painkillers during the Covid 19 pandemic.

Before COVID 19 pandemic, I have felt left alone with the pain.

During COVID 19 pandemic, I have felt left alone with the pain.

Before the pandemic I felt I had to bear the pain alone.

During the pandemic I felt I had to bear the pain alone.

0 1 2 3 4 5 6

n/a

**S001**

## Did the pain change due to the restricted lifestyle during COVID 19 pandemic?

I generally have no pain due to my endometriosis the pain got better

the pain was stable

the pain got slightly worse the pain got much worse

other thoughts (optional):

n/a

## Do you perceive your pain as more burdensome in the context of the pandemic?

I had no endometriosis-related pain during COVID 19 pandemic no

rather no rather yes yes

n/a

## Are you currently linked to a pain centre?

**S003**

- No, there has been no need recently
- No, due to the pandemic I couldn’t get an appointment
- No, due to the pandemic renounced my necessary appointment
- Yes, even before COVID 19 pandemic
- Yes, it got necessary during COVID 19 pandemic

alternative (optional)

n/a

**S012**

## How often did you take painkiller due to endometriosis related pain before COVID 19 pandemic?

daily

weekly monthly rarely

never

Keine Angabe

## How often did you take painkillers due to endometriosis related pain during COVDI 19 pandemic?

daily

weekly monthly rarely

never

n/a
